# Threshold, budget and deadline: beyond the discourse of climate scarcity and control

**DOI:** 10.1007/s10584-021-03185-y

**Published:** 2021-08-10

**Authors:** Shinichiro Asayama

**Affiliations:** National Institute for Environmental Studies, Tsukuba, Japan

**Keywords:** Temperature threshold, Carbon budget, Climate deadline, Scarcity, Post-politics, Emancipation

## Abstract

Since its inception, the Intergovernmental Panel on Climate Change (IPCC) has always been at the centre of the global climate debate. Its authoritative reports provide cultural resources for public understanding on the challenge of climate change. While the IPCC maintains its perception as a policy-neutral adviser, the IPCC in practice acts as a powerful *discursive* agent that guides policy debates in a certain direction by enacting influential scientific concepts. These concepts include three prominent metaphors—temperature threshold, carbon budget and climate deadline—that have been widely circulated across science, policy and advocacy. Three metaphors differ on ways in which the risk of climate change is expressed in terms of space and time. But they all constitute the discourse of *climate scarcity*—the cognitive view of that we have (too) little space and time to stay below a physical limit for avoiding dangerous climate change. This discursive construction of physical scarcity on climate change has significant political and psychological implications. Politically, the scarcity discourse has the risk of increasing a post-political tendency towards managerial control of the global climate (‘scarcity of politics’). Psychologically, however, scarcity has a greater risk of generating a ‘scarcity mindset’ that inhibits our cognitive capacity to imagine human life beyond managing physical scarcity. Under a narrow mindset of scarcity, the future is closed down to the ‘point of no return’ that, if crossed, is destined to be the end. To go beyond the scarcity discourse, a new discourse of *emancipation* has to be fostered. Climate change can be reframed not as a common single destination but as a predicament for actively reimagining human life. Such a narrative can expand our imaginative capacity and animate political action while embracing social losses.

## Introduction

The Intergovernmental Panel on Climate Change (IPCC) sits at the centre of the global climate debate across science, policy and advocacy. Since its establishment in 1988, the IPCC has evolved over time from something like an ad hoc, small panel of government-appointed scientists into a full-fledged 'international bureaucracy' (De Pryck 2018) that carries out multi-year cycle, comprehensive assessments about scientific knowledge on climate change. IPCC reports are now arguably considered the most authoritative voice of climate science. The IPCC has built its epistemic authority through painstaking efforts for the making of intergovernmental expert consensus (De Pryck 2021), which is guided by its own institutional mantra of so-called ‘policy-relevant but not policy-prescriptive’ advice. Such consensus-making and the guise of political neutrality renders to the IPCC a *symbolic* power—the power to make people see and believe, and hence act on, the world (Hughes 2015; see also Beck and Mahony 2018).

While carefully distancing itself from providing—or being perceived of providing—specific policy recommendations, the IPCC has a *performative* influence on shaping the political discourse about climate change. As Beck and Mahony (2017, 2018) argue, the technical elaboration of possible future pathways is an inseparable part of the ‘politics of anticipation’—bringing certain futures into a political reality. IPCC scenarios such as Representative Concentration Pathways (RCPs) or Shared Socioeconomic Pathways (SSPs) do not just represent possible states of the future but also emerge as discursive objects that can determine political actions in the present. These scenarios were created primarily as scientific heuristics for evaluating climate impacts and mitigation costs, but they also became political devices for justifying certain policy options (Hausfather and Peters 2020; Pielke and Ritchie 2021).

Like RCPs and SSPs, the IPCC has devised and formulated many scientific tools, concepts or frameworks for assessing knowledge about climate change. In some way, the IPCC can be seen as providing the cultural resources for communicating climate change in society. These scientific evaluative devices created for IPCC assessment are ‘boundary objects’ (Star and Griesemer 1989) that facilitate communication across different cultural spaces, travelling from scientific publications to policy documents to media coverage. They eventually become a primary vantage point from which people come to understand the challenge of climate change. IPCC assessment may not necessarily prescribe a specific policy, but it does fixate our way of knowing the climate problem to a specific viewpoint (Borie et al. 2021).

In this article, I argue that the IPCC should be seen as a powerful *discursive* agent that guides policy debates on climate change in a certain direction by enacting influential scientific metaphors. I pick up three examples of such concepts: temperature threshold, carbon budget and climate deadline. These scientific metaphors (and their iconic graphics) have been widely circulated around different ‘cultural circuits’ (Hall 1997) of climate change communication. Three metaphors differ on ways in which the risk of climate change is expressed in terms of space and time. But they all constitute what I call the discourse of *climate scarcity*—the cognitive view of that there is an upper limit to the level of warming that must not be exceeded to avoid dangerous climate change and that we have (too) little space (the allowable amount of CO_2_ emissions) and time (the date for reaching a temperature limit) to stay below the prescribed limit.

The implication of enacting the scarcity discourse is to generate a ‘scarcity of politics’. That is, the physical scarcity of temperature limit or carbon budget leaves little room for politics, being preoccupied by managerial control of the global climate. It may also lead to a ‘scarcity mindset’ that is to narrow down (or ‘tunnel’) our cognitive capacity to imagine the alternative, political possibilities of living with a changing climate. To go beyond the scarcity discourse, I argue, we should embrace more the discourse of *emancipation*.

## Three metaphors of scarcity: threshold, budget and deadline

Over the last few decades, international climate debates have centred on the question of how to define the ultimate objective of the 1992 United Nations Framework Convention on Climate Change (UNFCCC) to prevent ‘dangerous anthropogenic interference with the climate system’ in the form of a *quantitative* target. Among various metrics (such as greenhouse gas concentration, ocean heat content or sea-level rise), global average temperature emerged as the favoured indicator for quantifying the level of climate change. Scientifically, global temperature is considered the most suitable climate indicator because it serves as a useful intermediary between climate impacts assessment and greenhouse gas emissions projection (Knutti et al. 2016; Leemans and Vellinga 2017). From a communication perspective, the simplicity of temperature target is particularly seen as a merit, an easy way to communicate the complex challenge of climate change to the public (Schellnhuber et al. 2016; Morseletto et al. 2017).

The IPCC has played a pivotal role in elevating global temperature as a singular global index through which people know the problem of climate change and then act upon it (Hulme 2010). Here I delve into how the IPCC has helped enact three scientific metaphors—temperature threshold, carbon budget and climate deadline—all of which anchor the story of climate change to an index of global temperature. I chose these metaphors because they have become part of the general lexicon on climate change. It is also because the IPCC presented them with iconic visual images. Visual images carry symbolic messages that can transcend linguistic barriers, shaping the cultural discourse of climate change (O’Neill and Smith 2014). The graphics in IPCC reports are particularly powerful rhetorical devices since they are with the 'scientific veneer' of legitimacy (McMahon et al. 2016).

Metaphors enable us to see and understand one thing in terms of another. Nerlich and Jaspal (2012) argue that metaphors are *linguistic technology*, a tool we use to think and act with. The three metaphors discussed below are scientific concepts that were developed to summarise knowledge relevant to global temperature change. I call them scientific *metaphors* because they fulfil a metaphorical function to help people ‘see’ at what point climate change will enter dangerous territory and ‘know’ how close we are to that point. And these metaphors would generate the recognition of *scarcity* that (too) little space and time is left to stay within the physical limit for avoiding the dangerous consequences of climate change.

### Temperature threshold—the point of no return

The idea of a *temperature threshold* is rooted in long-standing efforts to define a stabilisation target for global temperature before further warming causes irreversible climatic impacts. Historically, the 2°C target had been long considered since 1990s a ‘safe limit’ to global temperature rise (Randalls 2010; Cointe et al. 2011; Jaeger and Jaeger 2011; Leemans and Vellinga 2017; Morseletto et al. 2017). The 2015 Paris Agreement and the subsequent release of the IPCC Special Report on Global Warming of 1.5°C (SR15) in 2018 changed the political context in which 1.5°C appeared a more preferable and safer target than 2°C (Tschakert 2015; Schleussner et al. 2016).

The IPCC has not prescribed (explicitly) the 1.5°C or 2°C target as a threshold above which warming enters into an intolerable zone. The IPCC has however played a critical role in enacting the idea of a temperature threshold through the development of the ‘reasons for concern’ (RFC) framework and its associated visualisation in the ‘burning embers’ diagram (Mahony and Hulme 2012; Mahony 2015; O’Neill et al. 2017; Zommers et al. 2020).

**Figure 1.**
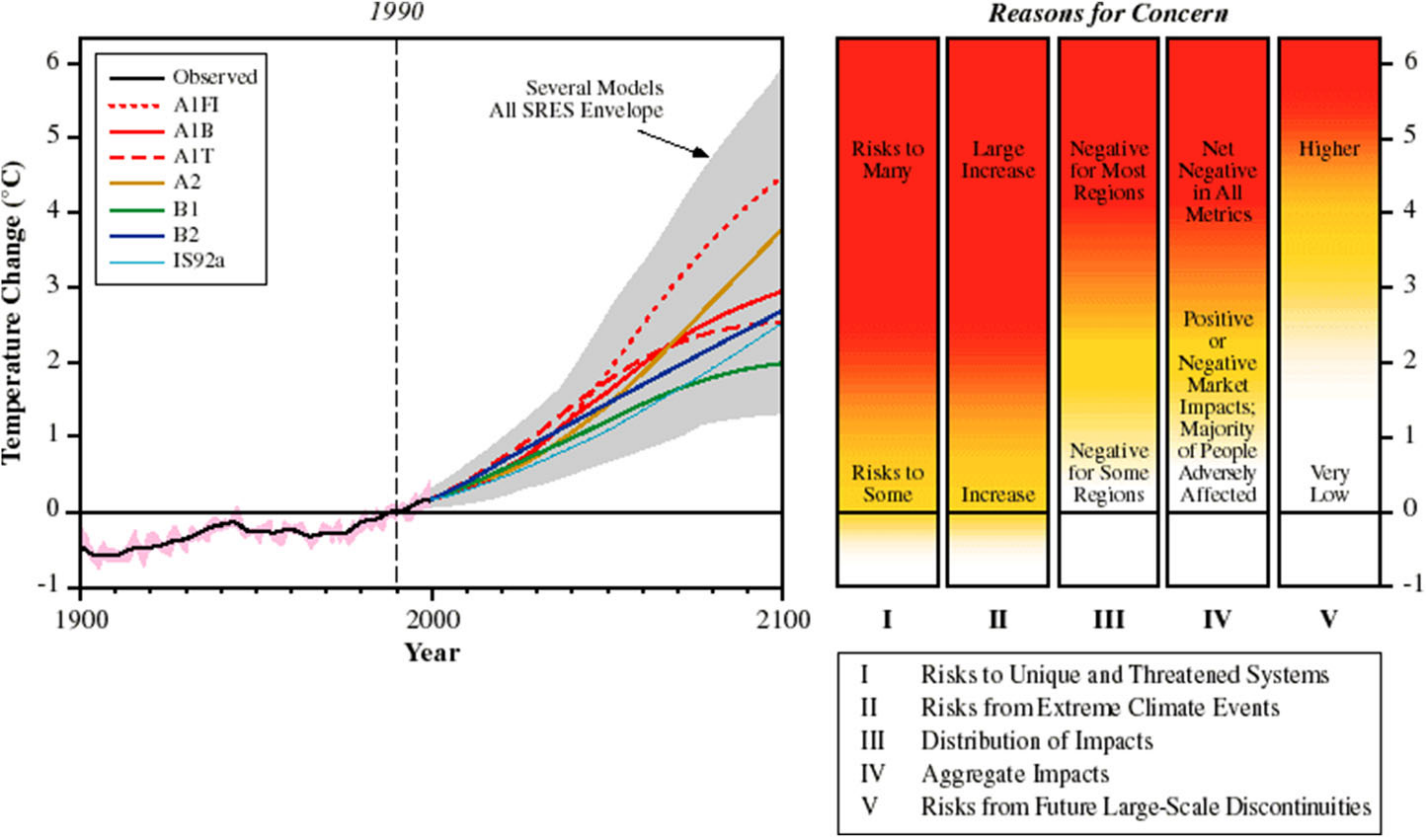
The burning embers diagram for five reasons for concern in the IPCC’s 2001 Third Assessment Report (TAR) (Source: IPCC 2001). While the original version of the burning embers appeared in greyscale in Chapter 19 of the Working Group (WG) II TAR, the first coloured edition was published in the WGII TAR Summary for Policy Makers (SPM). It is worth noting that the original figure in the TAR only showed temperature change relative to 1990 levels. It was during the approval of the Fifth Assessment Report (AR5) SPM that the updated version added a ‘second thermometer’ to illustrate temperature change since pre-industrial times (Tschakert 2015). Since the Paris Agreement, the default reference period shifted from 1990 levels to pre-industrial levels.

The RFC framework and the burning embers diagram were first introduced in the IPCC Third Assessment Report (TAR) in 2001 (see Fig.1). Their development was indeed meant to inform discussions about at what level of global temperature climate change might be considered dangerous (see Mahony and Hulme 2012; Mahony 2015). The RFC framework is to aggregate the global risks of climate change into five categories and evaluate the risk level of each category according to global temperature change (O’Neill et al. 2017). A different level of risk is then indicated by the colour scale of the burning embers, shifting gradually from white (undetectable) to yellow (moderate), red (high) and purple (very high). The burning embers diagram is arguably one of the most iconic figures of IPCC reports, which has been widely used to communicate the risk of anthropogenic climate change.

Importantly, the evaluation of risk levels for each RFC is based on expert judgement on the available scientific evidence. Since the TAR, the judgement of risk levels has been updated with each assessment cycle, reflecting new findings in climate science, which has generally shifted towards lower temperature over time (Zommers et al. 2020). For example, for the fifth type of RFCs (risks associated with large-scale singular events such as the collapse of the Greenland and West Antarctic ice sheets), the temperature at which the risk level becomes ‘high’ has fallen from ~5.5°C in the TAR to <2°C in the SR15 (see Zommers et al. 2020).

Despite that the colour of the burning embers was *deliberately* blurred to emphasise the ambiguity of boundaries between risk transitions from one level to the next, blurred colours were still interpreted by some as markers of absolute thresholds (Mahony 2015). Together with the popular use of the ‘tipping points’ metaphor in climate change communication (Russill and Nyssa 2009; van der Hel et al. 2018), the burning embers helped cement the notion of temperature threshold that, if crossed, will lead to abrupt and irreversible changes in the climate system. A prominent example of this is the latest call by scientists to declare a climate emergency in which the world is approaching a ‘global cascade of tipping points’ that might happen even at between 1°C and 2°C of warming (Lenton et al. 2019).

In a statement at the preparatory meeting for the 2019 Climate Action Summit, the UN Secretary-General António Guterres referred to the IPCC SR15 and described 1.5°C as the threshold beyond which the world will face ‘irreversible climate disruption’.^1^ His remark perhaps betokens wider public sentiment that the world is coming close to the ‘point of no return’.

### Carbon budget—the allowable limit

The view of 1.5°C or 2°C as a planetary threshold to avoid dangerous climate change has also become an anchoring point for understanding the mitigation challenge. While the idea of a temperature threshold can be used to convey what level of warming will be dangerous, it still needs to be translated into a more ‘actionable’ policy target to meet the temperature goal (Geden 2016). The scientific concept of a *carbon budget*—the finite amount of allowable CO_2_ emissions to stay below a given temperature—facilitates the discursive translation of a temperature goal into a mitigation target for reducing global CO_2_ emissions to net zero (Rogelj et al. 2019; Matthews et al. 2020).

The concept of carbon budget was established by the scientific discovery of the near-linear relationship between CO_2_-induced temperature change and cumulative CO_2_ emissions, often called the ‘transient climate response to cumulative carbon emissions’ or TCRE (MacDougall 2016). The linear property of TCRE allows a given temperature level to be linked *directly* to a fixed amount of cumulative CO_2_ emissions. This means that the challenge of meeting the 1.5°C or 2°C target can be quantified as the remaining amount of CO_2_ that can be emitted into the atmosphere. As a carbon budget is calculated by climate models, it effectively translates the subjective meaning of ‘danger’, which is based on the expert judgement applied in the RFC framework, into a more objective, quantitative figure derived from mathematical modelling. Thus, the number of carbon budgets may appear more scientifically credible than the blurry colour of the burning embers.

**Figure 2.**
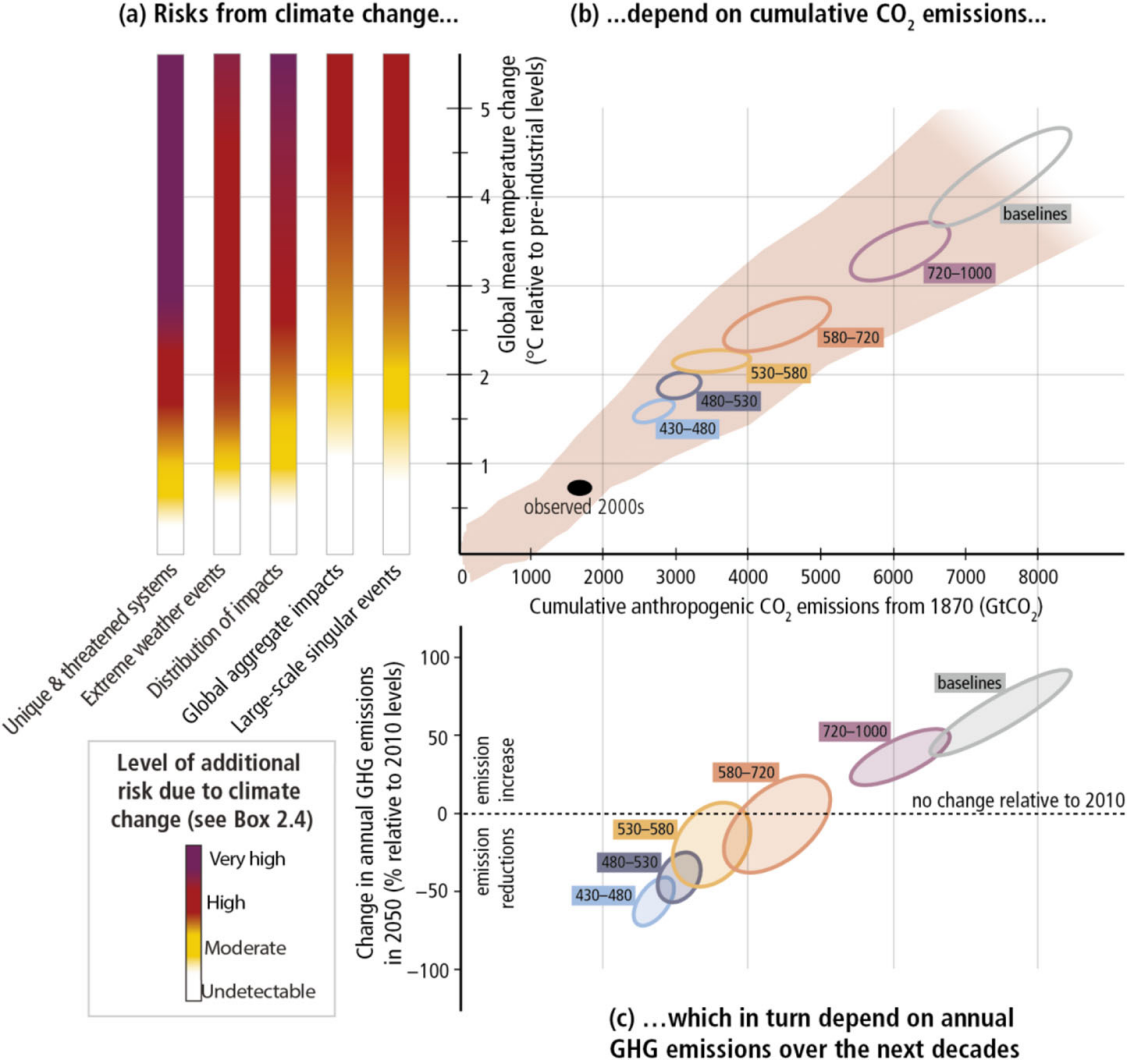
The relationship between risks from climate change, global temperature change, cumulative CO_2_ emissions and changes in annual greenhouse gas (GHG) emissions by 2050 (Source: IPCC 2014). The figure combines three graphics: the burning embers graphic (**a**); a graphic that shows the near-linear relationship between global temperature increase and cumulative CO_2_ emissions (**b**); and the projection of annual GHG emissions in 2050 relative to 2010 levels (**c**). The amount of cumulative CO_2_ emissions (carbon budget) fulfils the intermediary function of linking the risk assessment of climate impacts with the emission projection of climate mitigation.

The concept of the remaining carbon budget emerged around 2009 from modelling studies on the climate and carbon cycle system (see Lahn 2020). But it was the release of the IPCC’s Fifth Assessment Report (AR5) in 2013–2014 that firmly enacted the carbon budget as a new, central concept for guiding the climate policy debate (Lahn 2021). As Lahn (2021) noted, the IPCC provided an institutional infrastructure that enabled the group of scientists who first developed this concept to present it to policymakers with authority and legitimacy. The iconic figure in the Synthesis Report (SYR) of AR5 clearly shows how the idea of the carbon budget became the focal point connecting the risk assessment of climate change impacts to the emissions projection of climate change mitigation (see Fig.2).

Following the publication of AR5, the concept of the remaining carbon budget has been used widely in public discourse, adopted especially as the scientific basis for new climate activism, such as fossil fuel divestment and climate school strike (Lahn 2021; Strauch et al. 2020). For climate activists like Bill McKibben,^2^ the remarkably small size of the remaining carbon budget for the 1.5°C or 2°C target means more than a mere estimate by the modelling. It is often referred to as the ‘gospel of science’ that unbiasedly tells us how little amount of CO_2_ can be emitted in order to avert the climate crisis. The small number of carbon budget reveals the ‘inconvenient truth’ that a vast amount of fossil fuel reserves must stay in the ground as ‘unburnable carbon’ (Strauch et al. 2020). Importantly, the number can be trusted because it has been ‘authorised’ by IPCC assessment.

The story of the limited amount of carbon budget seems to capture, in particular, the mind of youth activists like Greta Thunberg. On many occasions, she displayed a sense of anger toward the current political atmosphere and stressed that ‘we need new politics, we need new economics where everything is based on a rapidly declining and extremely limited remaining carbon budget’ (Thunberg 2019). In the Thunberg’s eyes, carbon budget provides a *normative* frame of reference for reshaping our politics and economy based on the endless use of fossil fuels.

### Climate deadline—the marker of urgency

The concept of the carbon budget discursively translates a temperature limit into the ‘atmospheric disposal space’ for carbon emissions (Jakob and Hilaire 2015). The concept however enables a further conversion of the allowable amount of CO_2_ emissions into the time remaining to a *climate deadline*—the ‘due date’ for exhausting the remaining carbon budget at present levels of CO_2_ emissions (Asayama et al. 2019). The spatial scarcity of a finite carbon budget can be recalculated—through the modelling—into the time scarcity of a tight climate deadline.

The rhetorical use of deadlines in climate change communication has a long history (see Hulme 2020a). Perhaps the most famous kind of such deadline rhetoric is the Doomsday Clock that was created by the *Bulletin of the Atomic Scientists* in 1947. The origin of Doomsday Clock was a great fear of the threat posed by nuclear weapons, in particular a nuclear arms race between the United States and the Soviet Union. Using the minutes left until ‘midnight’ as the imagery of apocalypse, the clock was invented to warn the public about how close the world is approaching catastrophe, originally from nuclear weapons, but now including the danger caused by climate change. The remaining time to midnight is reset every year and decided by scientists and experts on the *Bulletin*’s board. The hands of the clock have no literal meaning; they are a metaphor—‘a reminder of the perils we must address if we are to survive on the planet’.^3^ Despite its metaphoric characteristic, the Doomsday Clock has become a powerful symbol that captures people’s minds and stokes their anxieties over an existential threat to humanity (Vuori 2010).

A similar sort of countdown clock is being set up by the Mercator Research Institute on Global Commons and Climate Change (MCC) in Germany. This Carbon Clock of the MCC shows how much time is left before the carbon budgets for the 1.5°C and 2°C targets are exhausted, counting down each second until the due date.^4^ Crucially, the calculated time on the clock is derived from the IPCC SR15’s estimate of the remaining carbon budget. Unlike the figurative nature of the *Bulletin’s* Doomsday Clock, the MCC’s Carbon Clock conveys a more literal sense of a deadline that is based not on the subjective judgement of experts but on a precise measurement estimated by the modelling. However, the intention behind setting up this Carbon Clock is similar to the Doomsday Clock—to warn the public how little time is left to avoid the worst effects of climate change. Both clocks thus serve the same *metaphorical* function of evoking a sense of urgency through the imagery of a ticking clock.

**Figure 3.**
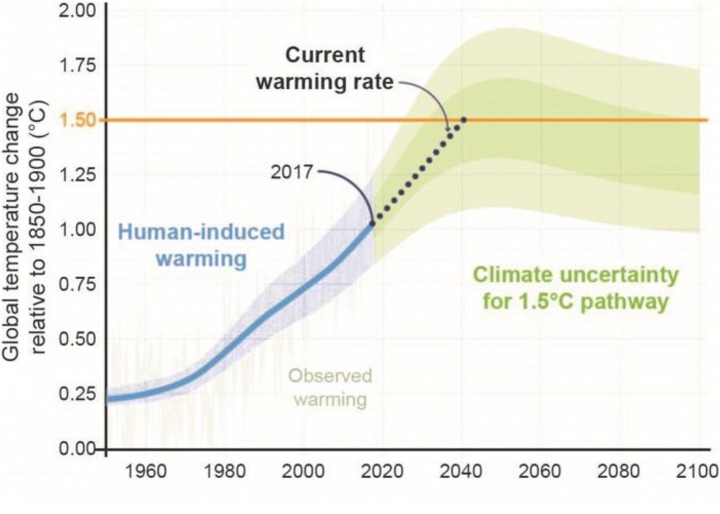
The estimated date by which global temperature will reach 1.5°C (Source: IPCC 2018). The 2018 IPCC Special Report on 1.5°C (SR15) estimated that human activities have caused approximately 1°C of warming above pre-industrial levels and that if the current pace of warming continues, warming will reach 1.5°C around 2040, within a likely range between 2030 and 2052.

The IPCC did not, of course, create such countdown clocks by itself. However, it can be argued that the IPCC helped create—though, rather unintendedly—the *political* conditions for that its findings were used as rhetorical devices for setting an imaginary deadline to urge immediate climate action (Asayama et al. 2019; Hulme 2020a). As one of the main conclusions of the SR15, the IPCC estimated that the remaining time to reach 1.5°C would be between 12 and 34 years from 2018 if the current rate of warming continues (IPCC 2018; see Fig.3). At the same time, the report also concluded that to limit global warming to 1.5°C, global CO_2_ emissions must decline by about 45% from 2010 levels by 2030, then reaching net zero around 2050. Soon after the release of SR15, this somewhat ‘dry’ statement was reinterpreted as some kind of an ‘alarm bell’ from scientists, warning us of how little time is left before it becomes too late to avert irreversible climate damage. The IPCC’s reference to the year 2030 made it appear as if a make-or-break deadline determining the fate of the climate.

Despite scientists’ criticisms that such rhetorical use of a deadline is misleading,^5^^,^^6^^,^^7^ the shorthand ‘we have only 12 years’ instantly became a common catchphrase among activists, and it was widely circulated in news reports and on social media (Boykoff and Pearman 2019; Bounegru et al. 2020). The widespread use of the 2030 deadline is also evidenced by the fact that prominent public figures, such as US congresswoman Alexandria Ocasio-Cortez^8^ and former US State Secretary John Kerry,^9^ made use of this rhetoric too. The 2030 deadline thus serves as a ‘marker of urgency’ signifying the need for radical mitigation in the present before it becomes ‘too late’ to stop dangerous climate change.

## Post-political control and scarcity mindset

As argued above, the IPCC has helped enact three scientific metaphors—temperature threshold, carbon budget and climate deadline—that have been influential in shaping how people see the challenge of climate change. IPCC assessments provide these metaphors with the veneer of scientific legitimacy (McMahon et al. 2016) and thereby enable them to circulate as boundary objects across science, policy and advocacy. They are powerful framing devices for climate change communication, all contribute to narrating the story of *physical scarcity*—that there is (too) little space and time to stay within a physical limit of global temperature change to avoid dangerous climate change.

Insomuch as this discursive construction of physical scarcity shapes our perception of the climate problem, it would also have significant implications for our views on politics and society. I argue that there would be at least two possible (negative) consequences of scarcity discourse—one political and one psychological—on our collective behaviours.

First, the discourse of physical scarcity could heighten the post-political managerial instincts of climate control (cf. Hulme 2010; Lövbrand et al. 2015). Given that scarcity means ‘having less than you feel you need’ (Mullainathan and Shafir 2013), managing scarcity requires being more attentive and efficient. Under conditions of scarcity, people cannot afford to waste limited resources. More prudent and effective management is crucial for preventing the overuse of scarce resource or the exceedance of physical limits. Here managing scarcity comes into conflict with democratic politics. It is because democratic decision-making often undergoes a slow process and hence may not suffice to produce decisive actions during a crisis. As scarcity discourse generates a growing sense of the climate crisis among the public, the legitimacy of the democratic mode of governance might be challenged in the name of ‘emergency’ (Hulme 2019; Nisbet 2019).

As such, scarcity discourse could potentially give rise to post-politicisation (‘scarcity of politics’) in which democratic politics are replaced by techno-managerial planning or governance by expert administrators (Swyngedouw 2013). Under post-political conditions, the policy debate is largely confined within the purview of managerial adjustments such as the choice of technologies and the timing of their implementation; normative and ideological questions are sidelined. This is how national governments in western democracy responded to the coronavirus pandemic crisis—and it *could* be repeated for addressing the climate crisis (Hulme et al. 2020).

The ‘symptoms’ of post-political managerialism, for example, manifest in the notion of ‘planetary stewardship’ promulgated by the group of Earth System scientists (Steffen et al. 2011, 2018). They emphasise the urgent need for effective planetary stewardship to maintain the stability of the Earth System and prevent the crossing of a threshold that will irreversibly lead to a dangerously hot planet. This sentiment of climate scientists resonates with the outcry from a new generation of youth climate movement where the phrase ‘follow the science’ is commonplace (Evensen 2019). What is alluded to in such trope is a deviation of the government’s political actions from the IPCC’s scientific advice. In the eyes of youth climate activists, the role of politics is relegated to simply following the *de facto* decisions made by science. Under scarcity, there appears no room for political fudging.

Second, scarcity discourse could have a profound psychological implication, that is, putting our cognitive mindset under scarcity (Shah et al. 2012; Mullainathan and Shafir 2013). According to Mullainathan and Shafir (2013), scarcity of any kind—be it a shortage of cash or a tight deadline—captures our attention and creates a *feeling* of scarcity. This scarcity mindset, they argue, changes how we think: it affects what we see, how we weigh our choices and even how we behave. Scarcity effectively changes how we interpret the world.

Mullainathan and Shafir (2013) show that a scarcity mindset provides both benefits and costs. On the one hand, scarcity makes people more attentive and efficient—it focuses our mind on what appears to matter most at the time. Mullainathan and Shafir (2013) called this benefit a ‘focus dividend’. Some could say that the 12-years deadline rhetoric raised a ‘focus dividend’ by increasing a sense of urgency of the climate crisis. On the other hand, scarcity comes at the cost of *tunnelling*—it leads people to neglect other important concerns that seem less urgent than the issue at hand. A strict budget or tight deadline creates its own narrow focus, and considerations other than managing scarcity fall outside the ‘tunnel’ (the scope of attention).

Consequently, a scarcity mindset forces *trade-off thinking* (Mullainathan and Shafir 2013). This is because physical limits make us feel that we have (too) little space or time to do everything—choosing one thing means not choosing something else. This trade-off thinking is evident in the framing of a zero-sum choice, by using the carbon budget concept, in fossil fuel divestment campaigns (Strauch et al. 2020). Divestment activists often argue that climate policy is about the choice of two incompatible futures: either a carbon-constrained world or a fossil-fuel development world.

The problem of this either-or framing is crowding out the cognitive space for imagining more politically nuanced, alternative pathways. Take for example the challenge of equitably managing the end of fossil fuel production (Lenferna 2018). While climate justice advocates often argue for leaving economically inefficient fossil fuels in the ground to stay within carbon budgets, there might be cases in which least developed countries seek to extract the least efficient fossil fuel reserves for meeting their human development needs. Lenferna (2018) pointed out that injustices may arise from pushing for the rapid phase-out of fossil fuels, following the logic of efficiency. Because this is an inherently value-laden and politically tough question, there is no simple yes-or-no answer. Framing the politics of ending fossil fuels in terms of zero-sum choices risks ignoring questions of equity pertaining to low-carbon energy transition.

## Towards the discourse of emancipation and care

Scarcity discourse may increase the risk of the post-political tendency towards controlling climate scarcity. From a communication perspective, however, scarcity discourse holds a more serious risk of inhibiting our cognitive capacity to imagine human life beyond the prevention of dangerous climate change. Under a narrow mindset of scarcity, we are forced to see climate change merely as the managerial problem of staying within a given physical limit. Despite the fact that climate change is an inherently complex and politically contentious challenge, our attention is fixated on hitting temperature targets or adhering to emissions budgets as some kind of ‘a common single destination’ (Hulme 2020b). There is no future imagined beyond these numbers. The scarcity discourse closes down our future until the ‘point of no return’—a point that, if crossed, the world will be destined to be the end. This is the *discursive limit* of communicating climate change through a lens of physical scarcity.

Late German sociologist Ulrich Beck (2009) argued that risk is socially constructed by the anticipation in the present of future catastrophes and that this nature of risk as an anticipated future catastrophe has the power to change the present world. Beck (2015) also challenged the focus of conventional policy discourse on finding solutions, arguing that such discourse is blind to the fact that climate change has *already* changed the world. He emphasised that talking about ‘bads’ would produce the new normative horizons of common goods—which he called the ‘hidden emancipatory side effect of global risk’ (Beck 2015). This is clearly evidenced by *Fridays for Future*, a climate justice movement led by the new generation. Young people are mobilised to walk out of school and onto the street not only by a sense of urgency of the climate crisis but due to moral and emphatic anger over the unfair treatment of the other (Antadze 2020). And yet, the slogan of this burgeoning youth activism is caught up in delivering the message of ‘listening to the science’ (Evensen 2019).

This is why we need a new language of *emancipation* to overcome the discursive limit imposed by a scarcity mindset. Crafting an emancipatory discourse means exercising our imaginative capacity to ‘see’ human life in a changing climate. Here, I want to give an account of three examples of such kind of discourse with emancipatory power.

First is the encyclical letter of Pope Francis, *Laudato Si’: On Care for Our Common Home*, issued in May 2015. The Pope’s encyclical was widely praised by the international climate community, partly because the Pope has moral authority and his voice can reach a wide range of people, including non-Christians. However, the most significant part of the Pope’s encyclical was that it framed climate change as a deeply moral issue rooted in our way of life and placed virtue and human dignity at the centre of climate discourse (Brulle and Antonio 2015; Hulme 2015; Jamieson 2015; O’Riordan et al. 2015). In particular, Pope Francis challenged dominant neoliberal views on consumerism (Carvalho 2015) and called for rethinking our ‘post-political attitude’ of that ‘there is no alternative to continuing our current growth-oriented, consumerist market economy’ (Brulle and Antonio 2015). Hulme (2015) argued that the encyclical was not so much about climate change; it was an invitation to the spiritual searching of ‘what it means to be human’ in an age of climate change.

This goes with the second example of emancipatory discourse: the *Green New Deal*, a radical policy proposal chiefly sponsored by US Representative Alexandria Ocasio-Cortez. Although the Green New Deal proposal arose from a sense of urgency fuelled by the 12-years deadline rhetoric, it went beyond the technicality of hitting a target and brought social and economic justice into the heart of climate policy debate. By encompassing a broad range of social welfare issues from a universal healthcare to affordable housing and a job guarantee, the Green New Deal is essentially about building the fair society with a decent life for all in the world of a changing climate. The symbolic power of the Green New Deal does not rest in its calculable political realism; rather it comes from the *boldness* of putting forth a radically progressive agenda that would have otherwise remained unimaginable (Klein 2019). This spirit of boldness made it possible for people to imagine a future in which ‘we can be whatever we have the courage to see’.^10^

The third example is a controversial essay written by an American author Jonathan Franzen in *The New Yorker* magazine.^11^ The article, titled *What If We Stopped Pretending the Climate Apocalypse Can Be Stopped?*, received heavy criticism from climate activists, probably because it could be read as an acceptance of defeat. By posing a provocative question, Franzen nevertheless expanded our imagination of what can be considered meaningful climate actions even *after* the world exceeds 2°C of warming. Such imaginative thinking enables us to see the things that had not been thought of as being related to climate change as the things that are deeply intertwined with—and thereby bringing new meanings into our social life. Rather than abandoning all hope, Franzen offered a *different* kind of hope—not the audacious kind embraced by Ocacio-Cortez, but a more humble and pragmatic kind of hope (see also Hulme 2020c). This is what a Norwegian psychologist Per Espen Stoknes (2015) called a *grounded hope*: ‘It’s grounded in our being, in our character and calling, not in some expected outcome’.

Taken together, these examples show the incalculable power of human ingenuity to imagine new possibilities in a predicament. Importantly, recognising—but not denying—climate change as a *human predicament* offers us to see the possibility of living with a changing climate (Hulme 2020c). From this perspective, climate change may be seen ‘unsolvable’, but it could still be a political and moral force to liberate the present from the legacy of a past that none of us chose and lead to the future of our choosing.

Creating an emancipatory discourse is also meant to shift our focus from *control* to *care*. It impels us to pay closer attention to societal losses that might inevitably arise from climate change (Barnett et al. 2016; Asayama et al. 2021). Building our sense of togetherness and ability to care better for the *unknown other* through empathy (Antadze 2019) will help us collectively come to terms with climate loss. Such a cultural narrative can animate political action while embracing social losses.

## Conclusion

For the nearly three decades since its inception, the IPCC has maintained, in principle, its own institutional mandate as a policy-neutral adviser. In practice, however, the IPCC has been acting as a powerful *discursive* agent that directs people into seeing the challenge of climate change from a ‘transcendental vantage point’ (Mahony 2015) or ‘view from nowhere’ (Borie et al. 2021; see also Shapin 1998). In particular, the IPCC helped fixate the story of climate change to the single metric of global mean temperature. As the IPCC becomes the global authority of climate science, 1.5°C or 2°C not only becomes a policy target but also comes to represent the collective psyche of our times.

In this article, I showed how the ostensibly policy-neutral objects of IPCC knowledge have been woven into the social fabric of cultural discourses, producing metaphors for ‘seeing’ where and when climate change might become dangerous. The IPCC thus is politically responsible not only for having helped enact the discourse of scarcity but also for cultivating a new discourse of emancipation to attenuate the risk of the scarcity mindset. However, the long-standing dominance of positivist disciplines—such as natural sciences and economics—in the IPCC’s knowledge assessment has marginalised the role of interpretative social sciences and the humanities (Hulme 2011). To foster an emancipatory narrative, the IPCC should engage in more meaningful ways with these interpretative disciplines.

Climate change is not just a scientific fact; it is also now a ‘social fact’ that shapes the public discourse of our time (Raman and Pearce 2020). It is now time for the IPCC to go beyond enacting the discourse of scarcity and offer a new emancipatory language for more imaginative conversations about climate change and our human life.

## Data Availability

No new data or model outputs were generated as part of this study.

## Footnotes

1 United Nations Secretary-General, ‘Secretary-General’s remarks to Climate Summit Preparatory Meeting’, 30 June 2019. https://www.un.org/sg/en/content/sg/statement/2019-06-30/secretary-generals-remarks-climate-summit-preparatory-meeting

2 Bill McKibben, ‘Global warming’s terrifying new math’, *Rolling Stones*, 19 July 2012. https://www.rollingstone.com/politics/politics-news/global-warmings-terrifying-new-math-188550/

3 https://thebulletin.org/doomsday-clock/faq/

4 https://www.mcc-berlin.net/en/research/co2-budget.html

5 Myles Allen, “Why protesters should be wary of ‘12 years to climate breakdown’ rhetoric”, *The Conversation*, 19 April 2019. https://theconversation.com/why-protesters-should-be-wary-of-12-years-to-climate-breakdown-rhetoric-115489

6 Andrew Freedman, ‘Climate scientists refute 12-year deadline to curb global warming’, *Axios*, 22 January 2019. https://www.axios.com/climate-change-scientists-comment-ocasio-cortez-12-year-deadline-c4ba1f99-bc76-42ac-8b93-e4eaa926938d.html

7 Edith Lederer, “UN climate chief warns current path leads to ‘catastrophe’”, *AP News*, 26 April 2019. https://apnews.com/article/united-nations-international-news-us-news-climate-a0baaad17de744ca875711d92e173442

8 John Bowden, “Ocasio-Cortez: ‘World will end in 12 years’ if climate change not addressed”, *The Hill*, 22 January 2019. https://thehill.com/policy/energy-environment/426353-ocasio-cortez-the-world-will-end-in-12-years-if-we-dont-address

9 “John Kerry says Earth has 9 years to avert the worst consequences of climate crisis: ‘There’s no faking it on this one’”, *CBS News*, 19 February 2021. https://www.cbsnews.com/news/climate-change-9-years-john-kerry/

10 Naomi Klein, ‘A message from the future with Alexandria Ocasio-Cortez’, *The Intercept*, 17 August 2019. https://theintercept.com/2019/04/17/green-new-deal-short-film-alexandria-ocasio-cortez/

11 Jonathan Franzen, ‘What if we stopped pretending the climate apocalypse can be stopped?’, *The New Yorker*, 8 September 2019. https://www.newyorker.com/culture/cultural-comment/what-if-we-stopped-pretending

## References

[CR1] Antadze N (2019). Who is the other in the age of the Anthropocene? Introducing the unknown other in climate justice discourse. Anthropol Rev.

[CR2] Antadze N (2020). Moral outrage as the emotional response to climate injustice. Environ Justice.

[CR3] Asayama S, Bellamy R, Geden O (2019). Why setting a climate deadline is dangerous. Nat Clim Chang.

[CR4] Asayama S, Emori S, Sugiyama M (2021). Are we ignoring a black elephant in the Anthropocene? Climate change and global pandemic as the crisis in health and equality. Sustain Sci.

[CR5] Barnett J, Tschakert P, Head L, Adger WN (2016). A science of loss. Nat Clim Chang.

[CR6] Beck U (2009). *World at Risk*.

[CR7] Beck U (2015). Emancipatory catastrophism: what does it mean to climate change and risk society?. Curr Sociol.

[CR8] Beck S, Mahony M (2017). The IPCC and the politics of anticipation. Nat Clim Chang.

[CR9] Beck S, Mahony M (2018). The IPCC and the new map of science and politics. Wiley Interdiscip Rev Clim Chang.

[CR10] Borie M, Mahony M, Obermeister N, Hulme M (2021). Knowing like a global expert organization: comparative insights from the IPCC and IPBES. Glob Environ Chang.

[CR11] Bounegru L, De Pryck K, Venturini T, Mauri M (2020). “We only have 12 years”: YouTube and the IPCC report on global warming of 1.5oC. First Monday.

[CR12] Boykoff M, Pearman O (2019). Now or never: how media coverage of the IPCC Special Report on 1.5C shaped climate-action deadlines. One Earth.

[CR13] Brulle RJ, Antonio RJ (2015). The Pope’s fateful vision of hope for society and the planet. Nat Clim Chang.

[CR14] Carvalho A (2015). The Pope’s encyclical as a call for democratic social change. Nat Clim Chang.

[CR15] Cointe B, Ravon P-A, Guérin E (2011). *2°C: The History of A Policy-Science Nexus*.

[CR16] De Pryck K (2018) *Expertise under controversy: the case of the Intergovernmental Panel on Climate Change (IPCC)*. PhD Dissertation, Sciences Po — Institut d’études politiques de Paris.

[CR17] De Pryck K (2021). Intergovernmental expert consensus in the making: the case of the Summary for Policy Makers of the IPCC 2014 Synthesis Report. Glob Environ Polit.

[CR18] Evensen D (2019). The rhetorical limitations of the #FridaysForFuture movement. Nat Clim Chang.

[CR19] Geden O (2016). An actionable climate target. Nat Geosci.

[CR20] Hall S (1997). *Representation: Cultural Representations and Signifying Practices*.

[CR21] Hausfather Z, Peters GP (2020). Emissions – the “business as usual” story is misleading. Nature.

[CR22] Hughes H (2015). Bourdieu and the IPCC’s symbolic power. Glob Environ Polit.

[CR23] Hulme M (2010). Problems with making and governing global kinds of knowledge. Glob Environ Chang.

[CR24] Hulme M (2011). Meet the humanities. Nat Clim Chang.

[CR25] Hulme M (2015). Finding the message of the Pope’s Encyclical. Environment.

[CR26] Hulme M (2019). Climate emergency politics is dangerous. Issues Sci Technol.

[CR27] Hulme M (2020). Is it too late (to stop dangerous climate change)? An editorial. Wiley Interdiscip Rev Clim Chang.

[CR28] Hulme M (2020). One earth, many futures, no destination. One Earth.

[CR29] Hulme M (2020). Climate change forever: the future of an idea. Scott Geogr J.

[CR30] Hulme M, Lidskog R, White JM, Standring A (2020). Social scientific knowledge in times of crisis: what climate change can learn from coronavirus (and vice versa). Wiley Interdiscip Rev Clim Chang.

[CR31] IPCC (2001). *Climate Change 2001: Impacts, Adaptation, and Vulnerability. Contribution of Working Group II to the Third Assessment Report of the Intergovernmental Panel on Climate Change*.

[CR32] IPCC (2014). *Climate Change 2014: Synthesis Report. Contribution of Working Groups I, II and III to the Fifth Assessment Report of the Intergovernmental Panel on Climate Change*.

[CR33] IPCC (2018) *Global Warming of 1.5°C. An IPCC Special Report on the impacts of global warming of 1.5°C above pre-industrial levels and related global greenhouse gas emission pathways, in the context of strengthening the global response to the threat of climate change, sustainable development, and efforts to eradicate poverty*. World Meteorological Organization, Geneva.

[CR34] Jaeger CC, Jaeger J (2011). Three views of two degrees. Reg Environ Chang.

[CR35] Jakob M, Hilaire J (2015). Unburnable fossil-fuel reserves. Nature.

[CR36] Jamieson D (2015). Why Laudato si’ matters. Environment.

[CR37] Klein N (2019). *On fire: the (Burning) case for a green new deal*.

[CR38] Knutti R, Rogelj J, Sedlácek J, Fischer EM (2016). A scientific critique of the two-degree climate change target. Nat Geosci.

[CR39] Lahn B (2020). A history of the global carbon budget. Wiley Interdiscip Rev Clim Chang.

[CR40] Lahn B (2021). Changing climate change: the carbon budget and the modifying-work of the IPCC. Soc Stud Sci.

[CR41] Leemans R, Vellinga P (2017). The scientific motivation of the internationally agreed “well below 2 °C” climate protection target: a historical perspective. Curr Opin Environ Sustain.

[CR42] Lenferna GA (2018). Can we equitably manage the end of the fossil fuel era?. Energy Res Soc Sci.

[CR43] Lenton TM, Rockström J, Gaffney O (2019). Climate tipping points — too risky to bet against. Nature.

[CR44] Lövbrand E, Beck S, Chilvers J (2015). Who speaks for the future of Earth? How critical social science can extend the conversation on the Anthropocene. Glob Environ Chang.

[CR45] MacDougall AH (2016). The transient response to cumulative CO2 emissions: a review. Curr Clim Chang Rep.

[CR46] Mahony M (2015). Climate change and the geographies of objectivity: the case of the IPCC’s burning embers diagram. Trans Inst Br Geogr.

[CR47] Mahony M, Hulme M (2012). The colour of risk: an exploration of the IPCC’s “burning embers” diagram. Spontaneous Gener A J Hist Philos Sci.

[CR48] Matthews HD, Tokarska KB, Nicholls ZRJ (2020). Opportunities and challenges in using remaining carbon budgets to guide climate policy. Nat Geosci.

[CR49] Mcmahon R, Stauffacher M, Knutti R (2016). The scientific veneer of IPCC visuals. Clim Chang.

[CR50] Morseletto P, Biermann F, Pattberg P (2017). Governing by targets: reductio ad unum and evolution of the two-degree climate target. Int Environ Agree Polit Law Econ.

[CR51] Mullainathan S, Shafir E (2013). *Scarcity: Why Having Too Little Means So Much*.

[CR52] Nerlich B, Jaspal R (2012). Metaphors we die by? Geoengineering, metaphors, and the argument from catastrophe. Metaphor Symb.

[CR53] Nisbet MC (2019). The trouble with climate emergency journalism. Issues Sci Technol.

[CR54] O’Neill SJ, Smith N (2014). Climate change and visual imagery. Wiley Interdiscip Rev Clim Chang.

[CR55] O’Neill BC, Oppenheimer M, Warren R (2017). IPCC reasons for concern regarding climate change risks. Nat Clim Chang.

[CR56] O’Riordan T, McGowan A, Hamann R (2015). The legacy of the Papal encyclical. Environment.

[CR57] Pielke RJ, Ritchie J (2021). Distorting the view of our climate future: the misuse and abuse of climate pathways and scenarios. Energy Res Soc Sci.

[CR58] Raman S, Pearce W (2020). Learning the lessons of Climategate: a cosmopolitan moment in the public life of climate science. Wiley Interdiscip Rev Clim Chang.

[CR59] Randalls S (2010). History of the 2°C climate target. Wiley Interdiscip Rev Clim Chang.

[CR60] Rogelj J, Forster PM, Kriegler E (2019). Estimating and tracking the remaining carbon budget for stringent climate targets. Nature.

[CR61] Russill C, Nyssa Z (2009). The tipping point trend in climate change communication. Glob Environ Chang.

[CR62] Schellnhuber HJ, Rahmstorf S, Winkelmann R (2016). Why the right climate target was agreed in Paris. Nat Clim Chang.

[CR63] Schleussner C-F, Rogelj J, Schaeffer M (2016). Science and policy characteristics of the Paris Agreement temperature goal. Nat Clim Chang.

[CR64] Shah AK, Mullainathan S, Shafir E (2012). Some consequences of having too little. Science.

[CR65] Shapin S (1998). Placing the view from nowhere: historical and sociological problems in the location of science. Trans Inst Br Geogr.

[CR66] Star SL, Griesemer JR (1989). Institutional ecology, “translations” and boundary objects: amateurs and professionals in Berkeley’s Museum of Vertebrate Zoology, 1907-39. Soc Stud Sci.

[CR67] Steffen W, Persson Å, Deutsch L (2011). The Anthropocene: from global change to planetary stewardship. Ambio.

[CR68] Steffen W, Rockström J, Richardson K (2018). Trajectories of the Earth System in the Anthropocene. Proc Natl Acad Sci.

[CR69] Stoknes PE (2015). *What we think about when we try not to think about global warming: toward a new psychology of climate action*.

[CR70] Strauch Y, Dordi T, Carter A (2020). Constraining fossil fuels based on 2 °C carbon budgets: the rapid adoption of a transformative concept in politics and finance. Clim Chang.

[CR71] Swyngedouw E (2013). The non-political politics of climate change. ACME An Int J Crit Geogr.

[CR72] Thunberg G (2019). *No one is too small to make a difference*.

[CR73] Tschakert P (2015). 1.5°C or 2°C: A conduit’s view from the science-policy interface at COP20 in Lima, Peru. Clim Chang Responses.

[CR74] van der Hel S, Hellsten I, Steen G (2018). Tipping points and climate change: metaphor between science and the media. Environ Commun.

[CR75] Vuori JA (2010). A timely prophet? The Doomsday Clock as a visualization of securitization moves with a global referent object. Secur Dialogue.

[CR76] Zommers Z, Marbaix P, Fischlin A (2020). Burning embers: towards more transparent and robust climate-change risk assessments. Nat Rev Earth Environ.

